# Rhizospheric *Bacillus amyloliquefaciens* Protects *Capsicum annuum* cv. Geumsugangsan From Multiple Abiotic Stresses *via* Multifarious Plant Growth-Promoting Attributes

**DOI:** 10.3389/fpls.2021.669693

**Published:** 2021-05-25

**Authors:** Elham Ahmed Kazerooni, Sajeewa S. N. Maharachchikumbura, Arjun Adhikari, Abdullah Mohammed Al-Sadi, Sang-Mo Kang, Lee-Rang Kim, In-Jung Lee

**Affiliations:** ^1^Department of Applied Biosciences, Kyungpook National University, Daegu, South Korea; ^2^School of Life Sciences and Technology, University of Electronic Science and Technology of China, Chengdu, China; ^3^Department of Crop Sciences, College of Agricultural and Marine Sciences, Sultan Qaboos University, Al-Khod, Oman

**Keywords:** 1-aminocyclopropane-1-carboxylate deaminase, pepper, endophyte, plant growth-promoting rhizobacteria, salinity, drought, phytoremediation

## Abstract

Plant growth-promoting rhizobacteria (PGPR) are beneficial microorganisms that can be utilized to improve plant responses against biotic and abiotic stresses. In this study, we investigated whether PGPR (*Bacillus amyloliquefaciens*) isolated from the endorhizosphere of *Sasamorpha borealis* have the potential to sustain pepper growth under drought, salinity, and heavy metal stresses. The bacterial strain was determined based on 16S rDNA and gyrB gene sequencing and characterized based on the following biochemical traits: nitrogen fixation; 1-aminocyclopropane-1-carboxylate deaminase activity; indole acetic acid production; inorganic phosphate, potassium, zinc, and silicon solubilization; and siderophore production. Various abiotic stresses were applied to 28-day-old pepper seedlings, and the influence of the PGPR strain on pepper seedling growth under these stress conditions was evaluated. The application of PGPR improved survival of the inoculated pepper plants under stress conditions, which was reflected by higher seedling growth rate and improved physiochemical traits. The PGPR-treated plants maintained high chlorophyll, salicylic acid, sugar, amino acid, and proline contents and showed low lipid metabolism, abscisic acid, protein, hydrogen peroxide contents, and antioxidant activities under stress conditions. Gene expression studies confirmed our physiological and biochemical findings. PGPR inoculation led to enhanced expression of XTH genes and reduced expression of *WRKY2, BI-1, PTI1*, and binding immunoglobulin protein (BiP) genes. We conclude that the PGPR strain described in this study has great potential for use in the phytoremediation of heavy metals and for enhancing pepper plant productivity under stress conditions, particularly those involving salinity and drought.

## Introduction

Plants are detrimentally affected by multiple environmental stimuli in the form of various abiotic and biotic stresses. Water deficiency and salinity are the prevalent environmental restraints that affect the morphological, physiological, biochemical, and molecular responses of plants and cause enormous losses in agriculture (Jaleel et al., 2007; Khan et al., 2019). Early responses of plants to these stresses lead to a slowdown of growth and photosynthesis, impair pigment production, increase nutrient deficiency, and reduce enzyme activity (Ali et al., 2014; Glick, 2014). In addition, soil pollution is also prevalent in different places. It is affected by industrial processes, cultivation practices, and human activities. These processes can lead to the spread of, for example, heavy metals, which can create severe ecological problems even at low levels (Wuana and Okieimen, 2011). There are other abiotic stresses that are more location specific, including frost (Keep et al., 2021; Simioniuc et al., 2021), ultraviolet radiation (Böttner et al., 2021; Navarro et al., 2021), and floods (Khan et al., 2020; Liu et al., 2021).

Plant growth-promoting rhizobacteria (PGPR) are occupant microorganisms in the rhizosphere that can enhance the positive results of phytoremediation. Once PGPR are applied in polluted soil, they improve the resistance of plants to heavy metals, expedite the turnover of nutrients, ameliorate the deleterious nature of some metals, boost plant tolerance against pests and pathogen assault, and improve soil structure (Khan and Bano, 2016; Karthik et al., 2017). Plant root exudates contain various metabolites and nutrients that are essential for nourishing rhizobacteria (Han et al., 2005; Babalola, 2010). PGPR provide a plethora of functions by which crop growth and yield can be improved. They have the capacity to fix atmospheric nitrogen, which is associated with the development of numerous growth-promoting plant hormones. Indole-3-acetic acid (IAA) producer strains enhance lateral root formation and improve root length, which assist plant to grow under drought conditions (Gupta and Pandey, 2019). Siderophore production is one of the main PGPR mechanisms to provide iron for plants in stressful situations. Previous studies showed that iron has a role in chlorophyll synthesis and maintain chloroplast structure (Rout and Sahoo, 2015). Additionally, PGPR comprise enzymes that act as mineral solubilizers and help maintain plant growth under stress conditions (Glick et al., 1998; Ma et al., 2009; Oves et al., 2013). The essential mechanisms by which PGPR improve plant growth during stress conditions include an increase in endogenous ethylene (ET) levels *via* 1-aminocyclopropane-1-carboxylate (ACC) deaminase enzyme activity, an increase in photosynthetic pigments, promotion of root development, rhizoremediation, and disease endurance (Belimov et al., 2005; Madhaiyan et al., 2006).

Phytoremediation is a promising technology with great capabilities for ameliorating soil pollution; it comprises the use of plants to “clean-up” environments by diminishing the toxicity, proportion, and diffusion of contaminants in soils (Liao and Chang, 2004). Plants can facilitate the removal of various contaminants, including pesticides and other chemicals, from soil and prevent the dispersion of pollutants *via* underground water reservoirs, wind, or rain from one region to another. This technology consists of two critical mutualistic components: plants and plant-associated microbes, which break down toxic substances into nontoxic substances (Saxena et al., 2005).

Pepper (*Capsicum annuum* L.) is one of the most economically important vegetable crops worldwide. It is commonly used as a fresh vegetable or spice owing to its pungent nature. Moreover, its fruit has major nutritional value and anticancer properties (Clark and Lee, 2016). However, this crop is highly susceptible to abiotic and biotic stresses, including salinity, drought, and pathogens; thus, plant yield is affected by such stresses, leading to economic losses (Dagdelen et al., 2004).

*Sasamorpha borealis* is an indigenous species that is highly prevalent throughout Korea (Lee and Yim, 2002). *S. borealis* is a strong clonal competitor, which successfully restrains other grassy plants and creates dense populations (Doležal et al., 2009). We speculated that PGPR isolated from *S. borealis* could enhance phytoremediation as well as salinity and drought tolerance in pepper plants under adverse environmental conditions. Therefore, this research was conducted to evaluate whether the application of PGPR could improve phytoremediation potential and help maintain the growth of salinity and drought-stressed pepper.

## Materials and Methods

### Collection and Isolation of Rhizospheric and Endophytic Bacteria

A fresh *S. borealis* (Hack.) Nakai plant was collected from Kyungpook National University, Daegu, South Korea. To isolate rhizospheric bacteria, a rhizospheric soil sample was processed based on the methods of Fischer et al. (2007). Endophytic bacteria present in the sample were isolated following a previously described method (Albdaiwi et al., 2019). Briefly, healthy roots were washed thoroughly under running tap water to remove adhering soil particles and then rinsed in double distilled water several times. Root samples were cut into small pieces and then surface sterilized in 70% ethanol for 1 min and 3% sodium hypochlorite solution for 10 min. Subsequently, they were rinsed in sterile distilled water three times. To validate root surface sterilization efficiency, an aliquot (20 μl) from the third wash solution was dispersed on Luria–Bertani (LB) medium and incubated at 28°C ± 2°C for 1 week. After that, sterilized roots (1 g) were aseptically crushed with a mortar and pestle using 10 ml of 1% NaCl and serial dilutions were prepared. Here, 1 ml of the root extract and the diluents were spread on LB agar medium. The plates were incubated at 28°C and surveilled up to 1 week for bacterial colony emergence. Morphologically dissimilar colonies of bacteria were designated and refined with streaking on LB agar medium. In total, 11 bacterial pure cultures were selected and preserved in corresponding growth medium with 25% glycerol at −20°C until further use. The selected colonies were named B1–B11, respectively.

### *In vitro* Assay for Plant Growth-Promoting Properties

All experiments were repeated three times, and each was conducted following a completely randomized design with three replications for each strain. The uninoculated medium served as a negative control.

### Citrate Utilization, Nitrogen Fixation, 1-Aminocyclopropane-1-Carboxylate Deaminase Activity, Indole-3-Acetic Acid, and Siderophore Production

A citrate test of bacterial strains was conducted by adopting the method of Simmons (1926). Citrate utilization ability was confirmed by the change in the color from green to blue. Siderophore production was examined using chrome azurol S (CAS) agar media following a previously described method (Alexander and Zuberer, 1991). The formation of a clear halo zone around the colonies was considered the result of siderophore production. Nitrogen-free bromothymol blue malate (NFb) medium was used for visual detection of nitrogen-fixing activities of bacterial strains (Baldani et al., 2014). A color change from green to blue indicated that the strain probably had nitrogen-fixing activity in the solid culture conditions. IAA production was determined following the methods of Sarwar and Kremer (1995). The appearance of a pink-red color confirmed IAA production by bacterial strains. The ACC deaminase activity of bacterial strains was assessed on sterile minimal Dworkin and Foster (DF) salts media containing 3-mM ACC (Sigma-Aldrich, United States) as a sole nitrogen source (Penrose and Glick, 2003). Bacterial strains multiplying on the ACC-supplemented plates were considered ACC deaminase producers (Ali et al., 2014).

### Potassium, Zinc, Phosphate, and Silicon Solubilization Assay

The ability of bacterial strains to solubilize inorganic phosphate was detected using Pikovskaya agar medium supplemented with tricalcium phosphate (Gaur, 1990). For determining potassium solubilization, the bacterial cultures were spot-inoculated onto Aleksandrov medium containing mica powder (Hu et al., 2006). Zinc solubilizing activity was determined in mineral salts agar medium supplemented with 0.1% zinc oxide (Venkatakrishnan et al., 2003). Si solubilization was assessed in the basal media supplemented with potassium alumina silicate (Sharma et al., 2011). The development of a clear zone around the bacterial colonies was considered to indicate solubilizing activity.

### *In vitro* Assay for Stress Tolerance in Response to Abiotic Stresses

The experiment was executed in triplicate with control and multiple test concentrations. The unmodified medium served as a control.

### Salinity Stress Tolerance

The intrinsic salt tolerance of bacterial strains was examined by observing their growth on the LB agar medium, which was supplemented with various NaCl concentrations (0.5%, 2.5%, 5%, 7.5%, and 10%). The plates were incubated for 72 h at 28°C ± 2°C (Barra et al., 2016).

### Drought Stress Tolerance

The drought endurance of strains was assessed in polyethylene glycol (PEG 6000)-supplemented LB agar medium. The strain growth was surveyed at 28°C ± 2°C for 24 h in LB agar media with different osmotic potentials (−0.05, −0.15, −0.3, −0.49, and −0.73 MPa) (Ali et al., 2014).

### Heavy Metal Stress Tolerance

The heavy metal tolerance of bacterial strains was examined on LB agar medium supplemented with varied cadmium (Cd), chromium (Cr), and nickel (Ni) concentrations ranging from 0.4 to 1.0 g L^–1^. The significant growth of bacterial strains in the presence of heavy metals during 72 h at 28°C ± 2°C was considered heavy metal tolerance (Cervantes-Vega et al., 1986).

### Extraction of Bacterial DNA and Phylogenetic Analysis

The selected bacterial strain (B11) was identified using 16S rDNA and the partial sequence of the gene encoding the subunit B protein of DNA gyrase (*gyrB*) sequencing and phylogenetic analysis. For this purpose, total genomic DNA was isolated as described by Chang et al. (2011). Polymerase chain reaction (PCR) assays were performed for 16S rDNA and *gyrB* regions using primers 27F/1492R and UP1F/UP2R (Supplementary Table 1). The preparation of the PCR reaction mixture and PCR amplification were completed following Ki et al. (2009) and Ahaotu et al. (2013). PCR products were purified and sequenced at SolGent Co., Ltd. (Daejeon, South Korea). The phylogenetic analysis of bacterial strain sequences was performed using RAxML GUI v.1.3 (Silvestro and Michalak, 2012).

### Effect of the Selected Bacterial Strain on Pepper Growth Under Drought, Salinity, and Heavy Metal Stresses

Based on the results of *in vitro* assays, the B11 bacterial strain was chosen and used for further assays. Specifically, the growth-promoting and phytoremediation effects of B11 on the growth of pepper under different abiotic stress conditions were assessed.

### Preparation of Bacterial Inoculum Suspension

A log phase culture of the B11 bacterial strain at the optical density (600 nm) was used to prepare the bacterial inoculum suspension. The selected bacteria were prepared in LB medium and maintained at 28°C in a shaker at 150 rpm for 24 h until late exponential growth phase. Afterward, the bacterial culture was centrifuged (8,000 × *g*, 10 min), and the supernatant was decanted. The obtained pellet was washed three times with sterile distilled water. Afterward, the obtained cell pellets were deferred in sterile distilled water (cell density of 10^7^–10^8^/ml), vortexed, and then used for plant treatment (50 ml/pot).

### Selection of Appropriate Concentration of Salt, Polyethylene Glycol, and Cadmium

Seeds of pepper (*C. annuum* cv. Geumsugangsan) were provided by Takii Korea Ltd. (Seoul, South Korea). They were surface sterilized in 70% ethanol and 1.5% sodium hypochlorite and tested for the efficiency of the sterilization process (Albdaiwi et al., 2019) and viability (Ke et al., 2018). Germinated seeds were planted in plastic pot trays (28 cm × 54 cm) filled with horticultural soil (Shinsung Mineral Co., Ltd., South Korea), with one seed planted per pot. Seeds were then grown in a climate chamber under the following conditions: 250 μmol photons m^–2^ s^–1^ PAR (photosynthetically active radiation), 65% relative humidity, 25°C ± 2°C, 16:8 h light:dark cycle, and daily watering of seedlings. Three-week-old seedlings with uniform sizes were transferred to pots (10 cm × 10 cm) and subjected to different treatments. All pepper seedlings were randomly divided into two groups: (i) normal control, grown with distilled water (50 ml/pot), and (ii) salinity treatment, irrigated with NaCl (0.5%, 1%, 1.5%, and 2.5%) (50 ml/pot). Each treatment contained three replicates of 15 plants each. Plants treated with each concentration were measured for various plant growth characteristics. Plant growth parameters such as plant height, root length, stem diameter, leaf length/width, number of leaves, chlorophyll content, plant fresh/dry weight, and root fresh/dry weight were measured after day 8 (8DAT) of treatment. Ultimately, 1% NaCl was found to be the optimal treatment concentration for use in further studies.

The optimal PEG treatment concentration was obtained *via* an experimental method described by Kaya and Doganlar (2019). In addition, we chose to expose pepper plants to 0.1% Cd stress.

### Plant Material and Growth Conditions

Experimental work was performed in a growth chamber at the Department of Applied Bioscience, Kyungpook National University. Plants were grown under the aforementioned conditions. In addition, sterilized pepper seeds (one seed/pot) were planted in plastic pot trays containing autoclave-sterilized horticultural soil (Shinsung Mineral Co., Ltd., South Korea). Seedlings of similar size (one seedling/pot) were selected after 3 weeks and then transferred to pots (10 cm × 10 cm). During the experiment, the temperature was maintained constantly at 25°C ± 2°C (day/night), with a relative humidity of 65% and a light intensity of approximately 200 μmol m^–2^ s^–1^. Soil moisture (70%), pH (∼7), and electrical conductivity (EC) (≤1.2) were recorded before the pot experiment; at the end of the experiment, soil samples from individual pots (300 g/pot) in each treatment were examined to again determine moisture, pH, and EC using a humidity tester (Model DM-5; Takemura Electric Works, Ltd., Tokyo, Japan) and conductivity meter (YSI Model 32, United States) (Supplementary Table 1).

### Experimental Design and Treatments

A 2 days after transplanting, 3-week-old seedlings were subjected to various treatments. They were divided into two groups: the normal control group, irrigated with sterile distilled water (50 ml/pot), and the bacterized group, irrigated with bacterial inoculum suspension (50 ml/pot). Each group was treated for 7 days, after which the groups of unbacterized and bacterized seedlings were subdivided into two groups containing an equal number of seedlings; this produced eight experimental groups, which are described in Table 1. Each treatment was performed twice in triplicate. The soil moisture level of each pot was monitored daily using a humidity tester (Model DM-5, as above). The pepper seedlings were exposed to selected abiotic stresses for 8 days, and sampling was performed at the end of each treatment. The harvested samples were either immediately used or rapidly deactivated in liquid nitrogen and stored at −80°C.

**TABLE 1:** Experimental work plan.

Symbol	Treatment
	
Cont	Irrigated with sterile distilled water
B11	Irrigated with PGPR B11
S	Irrigated with 1% NaCl
S+B11	Irrigated with 1% NaCl + PGPR B11
Dr	Irrigated with 10% PEG (−0.49 Mpa)
Dr+B11	Irrigated with 10% PEG (−0.49 Mpa) + PGPR B11
Cd	Irrigated with 0.1% Cd
Cd+B11	Irrigated with 0.1% Cd + PGPR B11

*PEG, polyethylene glycol; PGPR, plant growth-promoting rhizobacteria.*

### Physiobiochemical and Molecular Analyses of Plants

#### Plant Growth Characteristics and Chlorophyll Index Contents

To investigate the effect of each treatment on the seedlings, various plant growth parameters were measured. These parameters included plant height, root length, stem diameter, leaf length/width, plant fresh weight, and the number of leaves, all of which were recorded at 8DAT. A digital Vernier caliper and a ruler were used to measure stem diameter, leaf area (length and width), and plant height. In a preliminary salt screening experiment, chlorophyll content in leaves was examined using a portable CCM-300 Chlorophyll Content Meter (ADC BioScientific Ltd., Herts, United Kingdom).

#### Photosynthetic Pigments: Chlorophyll a, Chlorophyll b, and Carotenoid Content

Leaf chlorophyll [chlorophyll a (Chla), chlorophyll b (Chlb), and total chlorophyll (total Chl)] and carotenoid content were ascertained by spectrophotometric analysis of chemically extracted pigments (Arnon, 1949). Briefly, freeze-dried ground leaves were extracted in 80% ethanol at room temperature after centrifugation. Pigment absorption was then measured spectrophotometrically at 663, 645, and 480 nm (Thermo Fisher Scientific, Waltham, MA, United States).

#### Quantification of Pepper Phytohormones: Abscisic Acid and Salicylic Acid

The extraction and quantification of pepper abscisic acid (ABA) content were performed according to previously described methods (Qi et al., 1998; Kim et al., 2014). The resultant extract was dried with nitrogen gas (N_2_), and diazomethane (CH_2_N_2_) was added for methylation. ABA content was quantified by gas chromatography (GC)–mass spectrometry (MS) (Agilent 6890N Gas Chromatograph, Santa Clara, CA, United States). ThermoQuset software (Manchester, United Kingdom) was used to observe responses to ions (m/e of 162 and 190 for Me-ABA and 166 and 194 for Me-[^2^H_6_]-ABA).

To measure salicylic acid (SA) level in pepper plants, 0.1 g of freeze-dried sample was used as previously described (Enyedi et al., 1992; Seskar et al., 1998). Briefly, freeze-dried sample was extracted with methanol (90 and 100%) by centrifugation (12,000 rpm for 15 min at 4°C). The combined methanol extracts were vacuum dried, and the dried residue was dissolved in 5% trichloroacetic acid and centrifuged at 10,000 rpm for 10 min. The supernatant was partitioned with ethyl acetate:cyclopentane:isopropanol (49.5:49.5:1.0 ratio, v/v). The top layer of aqueous solution was dried and used for SA quantification using high-performance liquid chromatography (HPLC).

### Measurement of Amino Acid Content

Amino acid content was quantified using the method developed by Waqas et al. (2015). About 50 mg of freeze-dried leaves was hydrolyzed in the presence of hydrochloric acid (1 ml of 6-N HCl) for 24 h at 110°C. The extraction was then concentrated and vacuum dried at 80°C for 24 h. Afterward, the residue was diluted with deionized water (2 ml) and evaporated twice. The concentrated residue was then dissolved with hydrochloric acid (1 ml of 0.02-N HCl), and the mixture was passed through a 0.45-μm filter membrane. Finally, the solution was analyzed using a Hitachi L-8900 Amino Acid analyzer (Hitachi High-Technologies Corporation, Tokyo, Japan).

### Estimation of Leaf Protein and Sugar Content

Soluble protein content in the various treatments was quantified based on the methods of Ashraf and Iram (2005). The freeze-dried leaf samples (0.1 g) were ground and then mixed with a 1-ml phosphate buffer (50 mM, pH 7.0). The mixture was centrifuged at 10,000 rpm for 10 min at 4°C. Subsequently, the supernatant was collected and treated with an appropriate reagent, and the absorbance of each sample was assessed at 595 nm. Protein content was estimated in all enzymatic preparations using the Bradford method (Bradford, 1976), with bovine serum albumin as the standard.

Sugar content was estimated according to the defined method of Khan et al. (2018). Briefly, freeze-dried leaves were crushed, extracted using 80% ethanol, and then vacuum dried. The dried residue was redissolved in 1 ml of deionized water and passed through 0.45-μm nylon-66 syringe filters. Furthermore, the filtered samples were injected into an HPLC system (Millipore Co., Waters Chromatography, Milford, MA, United States).

### Determination of Enzymatic and Nonenzymatic Antioxidant Activity

The activity of antioxidant enzymes such as peroxidase (POD) and polyphenol oxidase (PPO) was analyzed using the method reported by Putter (1974). Superoxide dismutase (SOD) activity was investigated *via* the method of Sirhindi et al. (2016). To determine flavonoids, 1,1-diphenyl-2-picrylhydrazyl (DPPH) radical scavenging activities, and total polyphenols, samples were processed and analyzed following previously described procedures (Zheng and Wang, 2001; Barka et al., 2006; Wang et al., 2009; Li et al., 2016). The mixture activity and absorbance were measured at selected wavelengths using a Multiskan GO UV/Vis microplate spectrophotometer (Thermo Fisher Scientific, Waltham, MA, United States).

### Hydrogen Peroxide and Lipid Peroxidation

Following various treatments, hydrogen peroxide (H_2_O_2_) levels were determined as previously described (Jana and Choudhuri, 1982; Tsai et al., 2005). Frozen samples were freeze-dried and then ground with a pestle and mortar. The ground sample (0.3 g) was homogenized with 3 ml of ice-cold phosphate buffer [50 mM, 1 mM ethylenediaminetetraacetic acid (EDTA), 1% polyvinylpyrrolidone (PVP), pH 7.0] and centrifuged at 13,000 rpm for 20 min. The supernatant (2 ml) was mixed with 1 ml of 20% (v/v) H_2_SO_4_ containing 0.1% titanium chloride, and the mixture was centrifuged at 13,000 rpm for 20 min. Supernatant intensity was measured at 410 nm with a T60 UV-Vis Spectrophotometer (PG Instruments Ltd., Wibtoft, United Kingdom).

The level of lipid peroxidation was estimated through the thiobarbituric acid reaction, as described by López-Serrano et al. (2019). Malondialdehyde (MDA) content was calculated using its extinction coefficient. The amount of lipid peroxidation was shown as the level of MDA created per gram of tissue weight.

### Quantification of Macronutrients and Sodium and Cadmium Uptake in Bacterial Cells (B11) and Pepper Plants

The potential bacterial strain was grown in LB broth containing 1% NaCl, 10% PEG, and 0.1% Cd. The bacterial pellet was harvested and prepared for analysis as previously explained by Damodaran et al. (2013). For nutrient analysis of pepper plants, samples were first freeze-dried, and processed into a powder. Subsequently, the prepared samples were used to quantify Cd, sodium (Na), and nutrient (potassium, K; phosphorus, P; and calcium, Ca) uptake in pepper plants using inductively coupled plasma mass spectrometry (Optima 7900DV; Perkin-Elmer, United States). Treatments without bacterial inoculation were used to determine the initial concentrations of salt, heavy metals, and macronutrients. The removal efficiency of salt and Cd by the bacterial strain (B11) was calculated as follows: salt/Cd removal efficiency % = $\frac{\mathrm{C0}-\mathrm{Cs}}{\mathrm{C0}}\times 100$, where C0 and Cs represent the initial and final concentrations of salt and Cd, respectively.

### cDNA Synthesis and Real-Time PCR Analysis

Total RNA was extracted from the pepper leaves (8DAT) and used for cDNA synthesis and quantitative PCR (qPCR) following the procedure of Imran et al. (2018). Specifically, 1 μg of RNA was used to synthesize cDNA with a BioFACT RT-Kit (BIOFACT, Korea) following the manufacturer’s standard protocol. The synthesized cDNA was employed as a pattern in a two-step qRT-PCR reaction, which was performed to determine transcript quantity with an Illumina Eco system (Illumina, United States) (Supplementary Table 2).

### Statistical Analysis

The experiments were conducted in triplicate consecutively three times. The results were subjected to statistical analysis by ANOVA using R (version 4.0.3). A least significant difference (LSD) test (*p* < 0.05) was used to determine significant differences among treatments. The mean and standard deviation were estimated with Microsoft Excel 2017. Graphs were prepared with GraphPad Prism (version 6.01; San Diego, CA, United States).

## Results

### Bacterial Isolation and Taxonomic Characterization

**FIGURE 1:**
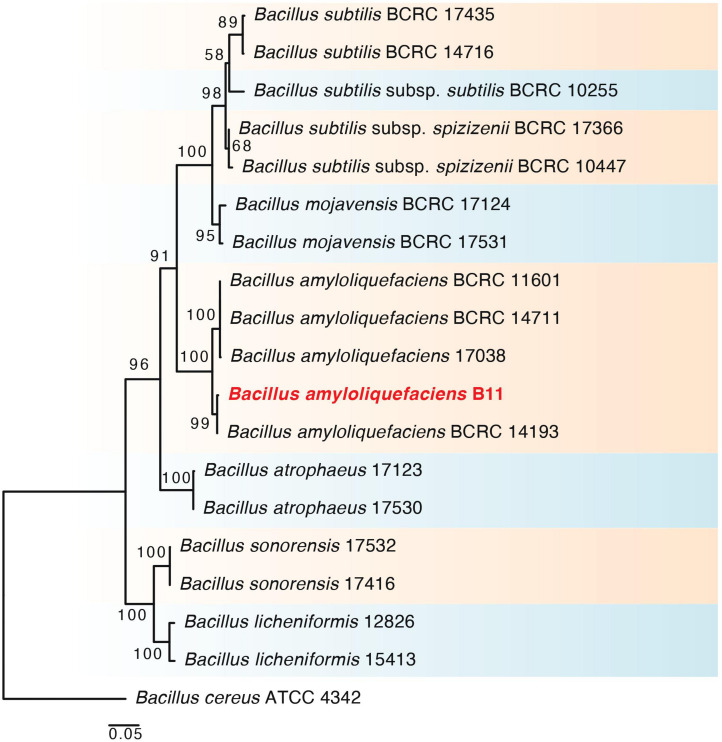
RAxML tree, based on partial 16s rDNA and *gyrB* nucleotide sequences and sequences from related *Bacillus* reference strains. The phylogram shows the relationship between the selected bacterial strain (B11) and the closely related taxa of *Bacillus amyloliquefaciens*. Bootstrap values (>50) are represented by numbers at the nodes based on 10,000 replications.

A total of 11 rhizobacteria were isolated from the rhizosphere and endosphere of *S. borealis* plants. The 16S rDNA and *gyrB* gene sequencing and phylogenetic analysis disclosed that the selected PGPR strain (B11) had a 100% similarity with the known sequences in GenBank and belonged to *Bacillus amyloliquefaciens*. In addition, phylogenetic analysis of the B11 strain using RAxML GUI v.1.3 software revealed its relevance to other strains of respective species (Figure 1). The 16S rDNA and *gyrB* gene sequences obtained in the present study have been submitted to the NCBI GenBank database and assigned the accession nos. MW599955 and MW602944, respectively.

### *In vitro* Plant Growth-Promoting Assay

The results of the *in vitro* growth-promoting assays in all strains are presented in Table 2 and Supplementary Figure 1. Most bacterial strains exhibited IAA and siderophore production, nitrogen fixation, and ACC deaminase activity (except B1). The strains B3–B8 did not show any citrate utilization activity. Our findings revealed variable solubilization activities: the strains B7, B10, and B11 were able to solubilize potassium, zinc, phosphate, and silicon.

**TABLE 2:** Growth-promoting and nutrient solubilization traits of 11 bacterial strains associated with *Sasamorpha* in this study.

Bacterial Isolates	Growth-promoting traits					Nutrient solubilization traits			
						Halo zone dm (mm)			
	IAA	ACC	Citrate	NF	Siderophore	P	Zn	Si	K
B1	+	−	+	+	+	5	4	7	−
B2	+	+	+	+	+	5	4	7	−
B3	+	+	−	+	+	10	−	−	−
B4	+	+	−	+	+	7	7	−	−
B5	+	+	−	+	+	10	5	−	−
B6	+	+	−	+	+	4	−	7	6
B7	+	+	+	+	+	6	10	7	5
B8	+	+	−	+	+	5	−	−	−
B9	+	+	+	+	+	10	5	−	−
B10	+	+	+	+	+	7	5	4	6
B11	+	+	+	+	+	10	10	8	13

*+ indicates positive, whereas − indicates negative. ACC, 1-aminocyclopropane-1-carboxylate; IAA, indole-3-acetic acid; NF, nitrogen fixation.*

### *In vitro* Stress Endurance in Response to Salt, Drought, and Heavy Metal

The bacterial strains were screened *in vitro* for their potential to tolerate salt, drought, and heavy metal stress conditions (Table 3). Most bacterial strains were able to tolerate salinity levels up to 10%, but some bacterial strains (B1, B2, B7, and B10) could not tolerate such levels. The strains B1 and B2 exhibited growth on LB media supplemented with only 0.5% and 2.5% NaCl. Similarly, the strains B7 and B10 could tolerate up to 5% salinity. The growth of strains B3 and B8 was inhibited on LB media supplemented with various concentrations of NaCl. In terms of their response to PEG-amended media, all bacterial strains were able to survive in drought conditions of “-0.05, −0.15, −0.3” MPa, as well as with “−0.49” and “−0.73” MPa osmotic stress (Table 3). Most bacterial strains showed visible growth on LB media supplemented with the tested Cr and Cd concentrations (Table 3). The specific results revealed different growth responses of the bacterial strains exposed to various Ni concentrations. Exceptionally, the strains B3 and B8 did not exhibit heavy metal resistance. The bacterial strain B11 indicated tolerance in all amended media; therefore, this strain was selected for further study (Table 3 and Supplementary Figures 2, 3).

**TABLE 3:** Salt, drought, and heavy metal tolerance abilities of bacterial strains associated with pepper in this study.

Bacterial isolates	Growth in NaCl (%)					Growth in PEG 6000 (MPa)					Growth in Heavy metal (g/L)								
	0.5	2.5	5	7.5	10	−0.05	−0.15	−0.3	−0.49	−0.73	Cr			Cd			Ni		
											0.4	0.8	1	0.4	0.8	1	0.4	0.8	1
B1	+	+	−	−	−	+	+	+	+	+	+	+	+	+	+	+	+	−	−
B2	+	+	−	−	−	+	+	+	+	+	+	+	+	−	−	−	−	−	−
B3	−	−	−	−	−	+	+	+	+	+	−	−	−	−	−	−	−	−	−
B4	+	+	+	+	+	+	+	+	+	+	+	+	+	+	+	+	−	−	−
B5	+	+	+	+	+	+	+	+	+	+	+	+	+	+	+	+	+	+	−
B6	+	+	+	+	+	+	+	+	+	+	+	+	+	+	+	+	+	+	−
B7	+	+	+	−	−	+	+	+	+	+	+	+	+	+	+	+	−	−	−
B8	−	−	−	−	−	+	+	+	+	+	−	−	−	−	−	−	−	−	−
B9	+	+	+	+	+	+	+	+	+	+	+	+	+	+	+	+	−	−	−
B10	+	+	+	−	−	+	+	+	+	+	+	+	+	+	+	+	−	−	−
B11	+	+	+	+	+	+	+	+	+	+	+	+	+	+	+	+	+	+	+

*+ indicates positive, whereas − indicates negative. PEG, polyethylene glycol.*

### Effects of Several Salt Concentrations on the Growth of Pepper Seedlings

The effect of salinity on pepper seedlings was investigated at NaCl concentrations of 0.5, 1, 1.5, and 2.5%. Treatment with the lowest NaCl concentration (0.5%) produced no remarkable change compared with the control plants (Supplementary Figure 4 and Supplementary Table 3). However, pepper plants treated with NaCl at 1% showed decreased plant height (33.12%), root length (19.35%), stem diameter (50%), leaf length (45.83%), leaf width (40.90%), plant fresh weight (66.66%), plant dry weight (50%), root fresh weight (55.55%), root dry weight (66.66%), and relative leaf number (27%) (*p* < 0.05) relative to these measures in control plants (Supplementary Figure 4 and Supplementary Table 3). Furthermore, irrigation with the highest level of NaCl (2.5%) caused a drastic reduction in plant growth parameters. Thus, 1% NaCl was the optimal concentration chosen for use in further studies.

### Pepper Seedling Response to Plant Growth-Promoting Rhizobacteria (B11) Inoculant Under Salinity, Drought, and Heavy Metal Stresses

#### Plant Growth Attributes

The influence of the selected bacterial strain (B11) on the growth of pepper seedlings without stress and under salinity, drought, and heavy metal stress conditions was evaluated in pot trials (Supplementary Figure 5). The negative effects of salinity, drought, and heavy metal stresses resulted in reduced growth parameters such as plant height, stem diameter, leaf area (length/width), total plant fresh weight, and number of leaves in relation to corresponding parameters in unstressed and uninoculated pepper plants (Table 4). However, plant height, stem diameter, leaf area (length/width), and fresh weight increased in PGPR-inoculated plants under salinity, drought, and heavy metal stress conditions. Specifically, plant height was improved by 14.77, 18.03, and 31.76% in the salt, drought, and Cd treatments in comparison with the corresponding heights of uninoculated and stressed plants (*p* < 0.05). Similarly, in bacterized plants, the total plant fresh weight was increased by 54.26%, 35.94%, and 33.26% in the salt, drought, and Cd treatments compared with the weights of the control stressed group of plants (Table 4).

**TABLE 4:** Effect of PGPR inoculation on pepper plant growth, chlorophyll a (Chla), chlorophyll b (Chlb), total chlorophyll (total Chl), and carotenoid contents under normal and stress conditions after 8 days of treatment (8DAT).

Treatment	Plant height	Root length	Stem diameter	Leaf length	Leaf width	Total Plant fresh weight	Chla	Chlb	Total Chl	Carotenoid	No. leaf
	(cm)	(cm)	(cm)	(cm)	(cm)	(g)	(μg/g FW)	(μg/g FW)	(μg/g FW)	(μg/g FW)	
**8DAT**											
Cont	20.6 ± 0.6a	18.0 ± 0.5e	0.3 ± 0.0a	9.4 ± 0.2a	5.5 ± 0.1a	12.11 ± 0.1b	25.9 ± 7.2a	9.9 ± 2.5f	104.4 ± 0.7b	1.3 ± 0.0a	16.0 ± 0.0a
B11	20.1 ± 0.2b	24.0 ± 0.5a	0.3 ± 0.0a	9.2 ± 0.5b	5.5 ± 0.4a	15.38 ± 0.6a	23.8 ± 6.0c	27.6 ± 7.1a	116.7 ± 1.6a	1.0 ± 0.5b	16.0 ± 0.0a
S	15.0 ± 1.0g	14.0 ± 0.5h	0.2 ± 0.0c	7.0 ± 0.5g	3.8 ± 0.4f	4.50 ± 0.7h	13.9 ± 3.0g	17.4 ± 4.0c	69.6 ± 6.3f	0.7 ± 0.9g	12.6 ± 0.5e
S+B11	17.6 ± 0.3d	18.8 ± 0.4d	0.3 ± 0.0a	7.8 ± 0.2e	4.6 ± 0.5d	9.84 ± 0.6e	21.2 ± 4.1d	18.5 ± 3.3b	97.3 ± 1.7c	0.9 ± 1.7d	14.3 ± 0.3b
Dr	15.1 ± 0.4f	14.5 ± 0.2g	0.2 ± 0.0c	7.5 ± 0.1f	4.1 ± 0.2e	6.38 ± 0.1g	13.59 ± 2.6h	12.4 ± 2.1e	68.33 ± 5.9g	0.72 ± 1.2f	13.0 ± 1.1d
Dr+B11	18.3 ± 0.1c	19.8 ± 0.6c	0.3 ± 0.0a	8.6 ± 0.2c	5.1 ± 0.3c	9.96 ± 0.1d	20.96 ± 3.7e	16.0 ± 2.3d	96.04 ± 1.5e	0.93 ± 2.0c	14.0 ± 0.5c
Cd	11.6 ± 2.3h	15.6 ± 0.4f	0.2 ± 0.0c	3.9 ± 2.0h	2.2 ± 1.1g	6.92 ± 0.4f	15.1 ± 2.6f	6.8 ± 0.2h	62.1 ± 1.4h	0.8 ± 1.4e	5.0 ± 2.6f
Cd+B11	17.0 ± 1.1e	22.0 ± 0.5b	0.3 ± 0.0a	8.1 ± 0.3d	5.2 ± 0.4b	10.37 ± 0.3c	23.9 ± 4.4b	9.6 ± 0.4g	97.1 ± 4.7d	1.0 ± 2.2b	13.0 ± 1.0d

*Treatments: control + water, control + plant growth-promoting rhizobacteria (PGPR), control + 1%NaCl, PGPR + 1% NaCl, control + 10% polyethylene glycol (PEG), PGPR + 10% PEG, control + 0.1% Cd, and PGPR + 0.1% Cd. Values show the means ± SE (n = 6) and significant differences at p < 0.05 [least significant difference (LSD) test].*

#### Chlorophyll and Carotenoid Content

Chlorophyll and carotenoid content was determined for pepper plants under both normal and stress conditions. Abiotic stresses are known to negatively affect the photosynthetic pigments of pepper plants. In the present study, plant pigments showed that Chla, Chlb, and carotenoid contents were increased in stressed plants inoculated with the bacterial strain (B11) compared with uninoculated stressed plants. Similarly, all the PGPR-treated stressed plants showed higher values of total chlorophyll content than the control stressed plants (Table 4). A considerable decrease in total chlorophyll content was observed in salt, drought, and heavy metal-stressed plants (33.33, 34.54, and 40.51%, respectively) in comparison with control plants. However, the PGPR inoculation was effective (*p* < 0.05) and resulted in a 28.46, 28.85, and 36.04% increase in total chlorophyll content under salt, drought, and heavy metal stress conditions, respectively, compared with the control stressed plants (Table 4).

#### Phytohormone (Abscisic Acid and Salicylic Acid) Regulation

**FIGURE 2:**
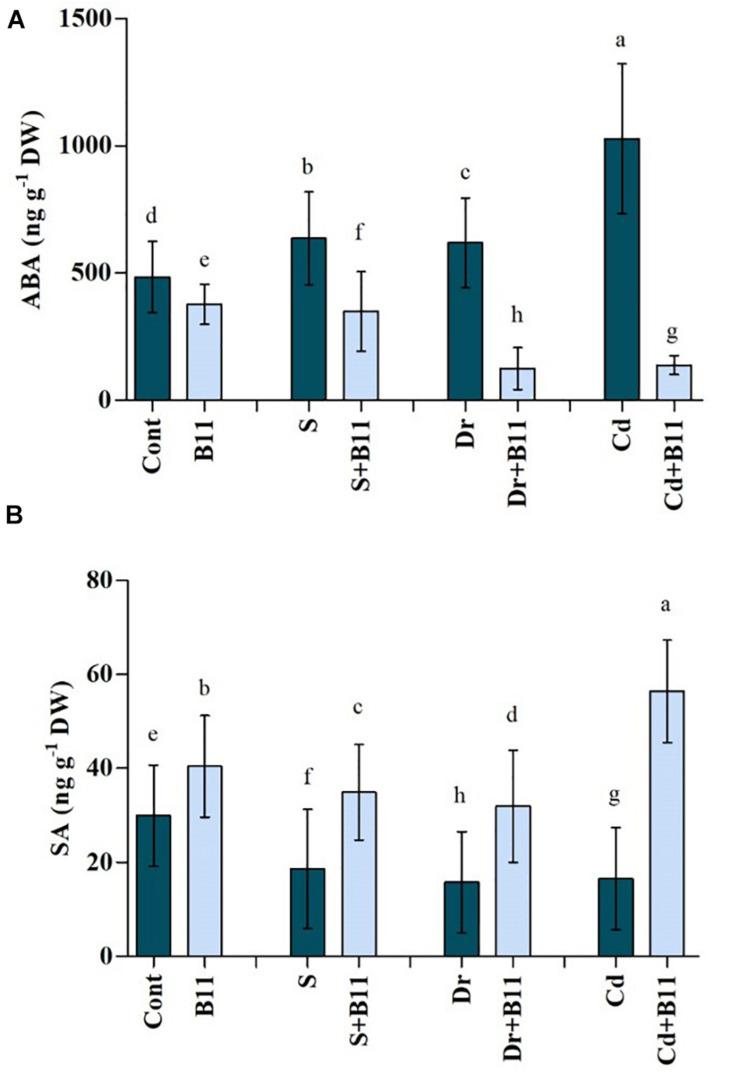
**(A)** Abscisic acid (ABA) and **(B)** salicylic acid (SA) content in leaves of pepper grown under normal and stress conditions and treated with plant growth-promoting rhizobacteria (PGPR) for 8 days (8DAT). Treatments: control + water, control + PGPR, control + 1% NaCl, PGPR + 1% NaCl, control + 10% polyethylene glycol (PEG), PGPR + 10% PEG, control + 0.1% Cd, and PGPR + 0.1% Cd. Values show the means ± SE (*n* = 3) and significant differences at *p* < 0.05 [least significant difference (LSD) test].

Endogenous ABA and SA contents were investigated over a period of 8 days to determine the influence of PGPR inoculation on pepper growth under control and abiotic stress conditions. Abiotic stresses induced an accumulation of ABA in pepper seedlings. However, PGPR treatment reduced ABA levels in pepper plants relative to the levels in control plants not subjected to abiotic stresses. Upon exposure to salinity, drought, and heavy metal stresses, PGPR-inoculated plants exhibited significantly reduced content of ABA, i.e., 45.15, 79.92, and 86.52%, respectively, compared with the content of uninoculated stressed plants (Figure 2A).

Compared with stressed plants, uninoculated plants showed a decrease in SA concentrations, which were reduced by 37.70% with salinity, 47.42% with drought, and 44.64% with heavy metals relative to the SA concentrations in control plants. Pepper seedlings inoculated with PGPR for 8 days, however, showed remarkable 46.55%, 50.70%, and 70.61% increases in SA content under salt, drought, and heavy metal stresses, respectively, compared with uninoculated stressed plants (Figure 2B). Indeed, our results suggested that PGPR inoculation resulted in enhanced SA content in pepper seedlings with or without stress.

#### Free Amino Acid Content

Eighteen amino acids with different concentrations were detected in pepper seedlings (Table 5). Over 8 days, salinity, drought, and heavy metal stresses increased amino acid content in pepper seedlings compared with seedlings under normal conditions. Proline content increased by 71.90%, 64.10%, and 58.83% in salinity-, drought-, and heavy metal-stressed plants, respectively. Similarly, plants with PGPR showed an increase in proline content. Additionally, 8 days after the application of PGPR in stressed plants, glutamic acid was found at the highest levels, whereas cystine and methionine were found at the lowest level in pepper seedlings subjected to normal and stress conditions (Table 5). These findings indicate that PGPR inoculation improved amino acid content under both stress and normal conditions.

**TABLE 5:** Effect of PGPR inoculation on the amino acid content of pepper plants grown under normal and stress conditions after 8 days of treatment (8DAT).

Amino acid	Treatment							
mg/g	Cont	B11	S	S+B11	Dr	Dr+B11	Cd	Cd+B11
**8DAT**								
Asp	13.76 ± 0.0h	23.13 ± 0.0b	18.69 ± 0.0f	20.53 ± 0.0c	16.16 ± 0.0g	23.48 ± 0.0a	19.84 ± 0.0e	20.25 ± 0.0d
Thr	6.91 ± 0.0h	12.08 ± 0.0a	10.01 ± 0.0e	10.75 ± 0.0c	8.18 ± 0.0g	11.88 ± 0.0b	8.55 ± 0.0f	10.04 ± 0.0d
Ser	6.30 ± 0.0h	14.22 ± 0.0a	11.03 ± 0.0d	11.85 ± 0.0c	7.0 ± 0.0g	13.19 ± 0.0b	8.53 ± 0.0f	10.84 ± 0.0e
Glu	18.72 ± 0.0h	33.06 ± 0.0d	26.20 ± 0.0f	31.0 ± 0.0e	22.40 ± 0.0g	33.70 ± 0.0c	37.37 ± 0.0b	48.50 ± 0.0a
Gly	8.81 ± 0.0h	14.86 ± 0.0a	12.62 ± 0.0e	13.46 ± 0.0c	10.02 ± 0.0g	14.49 ± 0.0b	11.66 ± 0.0f	13.44 ± 0.0d
Ala	9.35 ± 0.0h	16.09 ± 0.0a	13.28 ± 0.0e	13.94 ± 0.0c	11.46 ± 0.0f	16.02 ± 0.0b	11.10 ± 0.2g	13.82 ± 0.0d
Cys	1.09 ± 0.0h	2.15 ± 0.0e	2.19 ± 0.0d	2.57 ± 0.0b	1.66 ± 0.0g	1.81 ± 0.0f	2.32 ± 0.0c	5.29 ± 0.0a
Val	7.56 ± 0.0h	13.75 ± 0.0a	11.06 ± 0.0e	12.09 ± 0.0c	9.38 ± 0.0f	12.64 ± 0.0b	9.36 ± 0.0g	11.27 ± 0.0d
Met	1.10 ± 0.2h	2.30 ± 0.0b	2.31 ± 0.0a	2.03 ± 0.0d	2.0 ± 0.0e	2.10 ± 0.0c	1.67 ± 0.0g	1.97 ± 0.0f
Ile	6.34 ± 0.0h	11.13 ± 0.0a	8.93 ± 0.0e	9.87 ± 0.0c	7.59 ± 0.0f	10.14 ± 0.0b	7.45 ± 0.0g	9.31 ± 0.0d
Leu	13.91 ± 0.0h	23.92 ± 0.0a	19.59 ± 0.0e	20.80 ± 0.0c	16.77 ± 0.0f	23.17 ± 0.0b	15.76 ± 0.0g	20.06 ± 0.0d
Tyr	4.41 ± 0.0h	7.56 ± 0.0a	6.89 ± 0.0d	7.10 ± 0.2c	5.29 ± 0.0g	7.23 ± 0.0b	5.63 ± 0.0f	6.65 ± 0.0e
Phe	8.03 ± 0.0h	13.82 ± 0.0a	11.32 ± 0.0e	12.22 ± 0.0c	9.68 ± 0.0g	13.47 ± 0.0b	9.97 ± 0.0f	11.89 ± 0.0d
Lys	11.99 ± 0.0h	20.46 ± 0.0a	16.36 ± 0.0e	18.35 ± 0.0c	13.57 ± 0.0g	19.7 ± 0.0b	15.14 ± 0.0f	17.52 ± 0.0d
NH3	1.37 ± 0.0h	3.73 ± 0.0e	2.66 ± 0.0f	4.61 ± 0.0c	1.81 ± 0.0g	4.10 ± 0.2d	4.66 ± 0.0b	4.69 ± 0.0a
His	3.46 ± 0.0h	6.12 ± 0.0a	4.95 ± 0.0f	5.59 ± 0.0c	4.06 ± 0.0g	6.09 ± 0.0b	5.02 ± 0.0e	5.50 ± 0.0d
Arg	7.97 ± 0.0h	14.67 ± 0.0a	12.08 ± 0.0d	13.62 ± 0.0c	9.56 ± 0.0g	13.87 ± 0.0b	9.57 ± 0.0f	11.60 ± 0.0e
Pro	2.74 ± 0.0h	5.78 ± 0.0e	7.10 ± 0.0c	9.74 ± 0.0a	3.32 ± 0.0g	7.63 ± 0.0b	3.67 ± 0.0f	6.65 ± 0.0d

*Treatments: control + water, control + plant growth-promoting rhizobacteria (PGPR), control + 1% NaCl, PGPR + 1% NaCl, control + 10% polyethylene glycol (PEG), PGPR + 10%PEG, control + 0.1% Cd, and PGPR + 0.1% Cd. Values show the means ± SE (n = 6) and significant differences at p < 0.05 [least significant difference (LSD) test].*

#### Soluble Protein and Sugar Content

**FIGURE 3:**
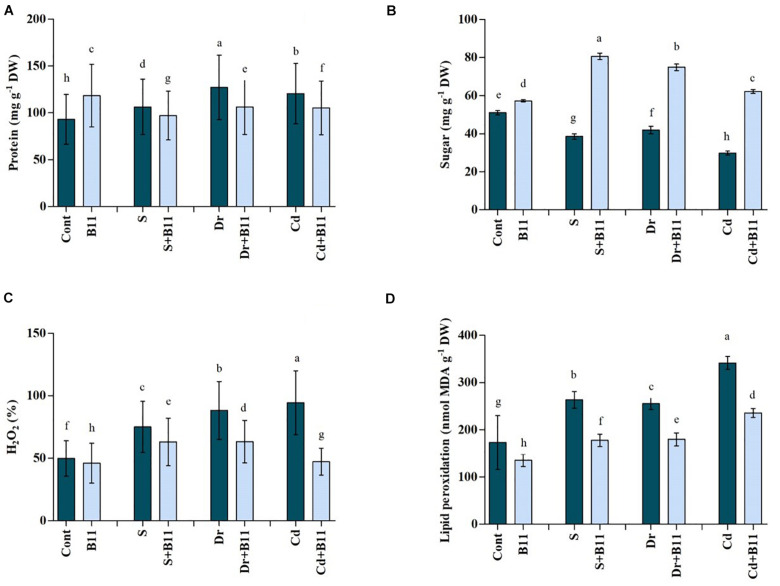
(**A**) Protein, (**B**) H_2_O_2_, (**C**) sugar, and (**D**) malondialdehyde (MDA) content in leaves of pepper grown under normal and stress conditions and treated with plant growth-promoting rhizobacteria (PGPR) for 8 days (8DAT). Treatments: control + water, control + PGPR, control + 1% NaCl, PGPR + 1% NaCl, control + 10% polyethylene glycol (PEG), PGPR + 10% PEG, control + 0.1% Cd, and PGPR + 0.1% Cd. Values show the means ± SE (*n* = 3) and significant differences at *p* < 0.05 [least significant difference (LSD) test].

Protein content increased in stressed seedlings over 8 days. Under normal conditions, the plant protein level increased by 21.45% upon PGPR inoculation compared with protein levels in uninoculated plants. The protein production rate was increased by 12.58%, 26.80%, and 22.77% under salinity, drought, and heavy metal stresses, respectively, when compared with protein production in unstressed plants (*p* < 0.05) (Figure 3A). Conversely, reductions in protein production were detected in PGPR-treated plants when they were exposed to abiotic stresses.

In the leaves of pepper plants, a substantial reduction in sugar content was observed after exposure to stress conditions (Figure 3B). Furthermore, sugar content decreased clearly in response to salt (24.31%), drought (17.77%), and heavy metal (41.43%) stresses when compared with the sugar content of plants under normal conditions (Figure 3B). The best outcomes were obtained when plants were treated with PGPR, which resulted in an increase in sugar content by 52.10%, 43.98%, and 51.93% under salt, drought, and heavy metal stress conditions relative to the sugar content of stressed plants alone (Figure 3B).

#### H_2_O_2_ and Malondialdehyde Content

Drought, salinity, and heavy metal stresses caused significant changes in H_2_O_2_ content in pepper plants (Figure 3C). H_2_O_2_ content was increased by 33.59%, 43.52%, and 47.20% under salinity, drought, and heavy metal stresses, respectively, compared with the H_2_O_2_ content in control plants. However, the application of PGPR was effective in reducing H_2_O_2_ content in stressed plants; maximum decreases of 16.05%, 28.23%, and 49.87% in H_2_O_2_ content were noted in PGPR-inoculated plants under salinity, drought, and heavy metal stresses, respectively (*p* < 0.05).

As shown in Figure 3D, stress conditions enhanced MDA production in untreated pepper plants; MDA content increased by 34.32%, 32.27%, and 49.37% under salt, drought, and heavy metal stress conditions, respectively. Compared with uninoculated plants, the reduction in MDA content in PGPR-treated plants was approximately 32.49% under salt, 29.71% under drought, and 31.05% under heavy metal stress conditions (*p* < 0.05).

### Antioxidant Content

**FIGURE 4:**
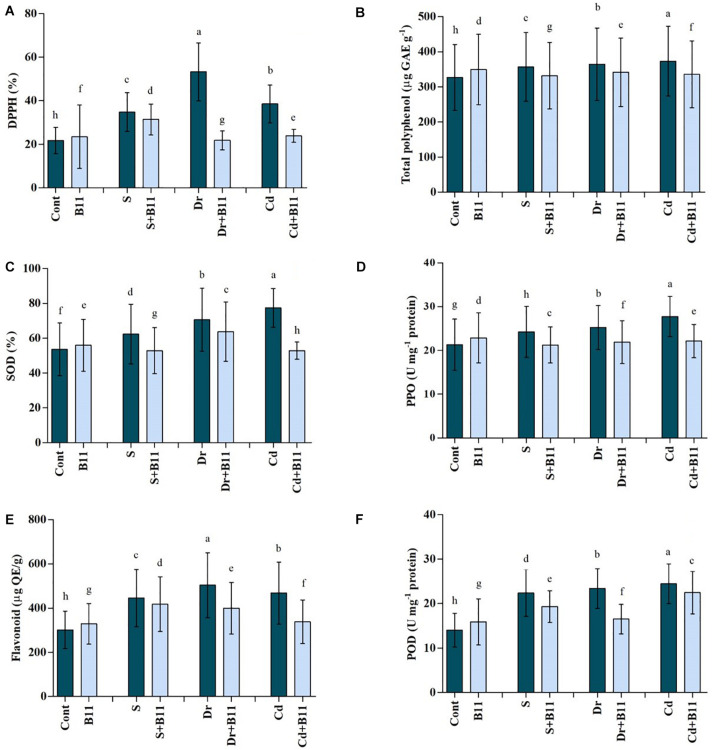
Antioxidant content [DPPH, **A**; total polyphenol, **B**; superoxide dismutase (SOD), **C**; polyphenol oxidase (PPO), **D**; flavonoids, **E**; and peroxidase (POD), **F**] of pepper leaves grown under normal and stress conditions and treated with plant growth-promoting rhizobacteria (PGPR) for 8 days (8DAT). Treatments: control + water, control + PGPR, control + 1% NaCl, PGPR + 1% NaCl, control + 10% polyethylene glycol (PEG), PGPR + 10% PEG, control + 0.1% Cd, and PGPR + 0.1% Cd. Values show the means ± SE (*n* = 3) and significant differences at *p* < 0.05 [least significant difference (LSD) test].

1,1-diphenyl-2-picrylhydrazyl content in pepper seedlings increased under stress conditions, whereas DPPH content was reduced in PGPR-treated seedlings exposed to all stresses. Specifically, PGPR inoculation contributed to 9.73%, 59.06%, and 37.99% reductions in DPPH content under salinity, drought, and heavy metal stresses relative to the DPPH detected in uninoculated stressed plants (Figure 4A). Furthermore, total polyphenol content was slightly enhanced under stress conditions, but it decreased under these conditions upon PGPR inoculation (Figure 4B).

Enhanced SOD activity was observed in pepper plants under stress conditions; however, SOD activity was lower in PGPR-treated plants subjected to the salinity (15.25%), drought (9.62%), and heavy metal (31.83%) stresses when compared with that of untreated stressed plants (Figure 4C).

Similar trends were observed with POD, PPO, and flavonoid activities, which increased distinctly under stress conditions. However, PGPR treatment reduced POD, PPO, and flavonoid content under stress conditions; for instance, their activities decreased (POD, 13.69%; PPO, 12.31%; flavonoid, 6.20%) in PGPR-inoculated seedlings subjected to salt stress (*p* < 0.05) (Figures 4D–F).

### Macronutrient, Sodium, and Cadmium Content in Plant and Bacterial Cells

To determine the effect of the PGPR inoculant on the macronutrient state of pepper plants and its detoxifying role, five elements, i.e., Ca, K, P, Na, and Cd, were examined (Table 6). Under normal conditions, an increase was detected in the concentrations of K and P in plants inoculated with PGPR relative to the respective concentrations in control plants. Furthermore, Ca content was higher in inoculated plants than in control plants under normal conditions. Compared with the stressed plants and the PGPR-inoculated plants, plant macronutrients were regulated in inoculated stressed plants, which showed increases in K and P concentrations and a decrease in the concentration of Ca under stress conditions. Moreover, PGPR significantly reduced the accumulation of Na and Cd in plants under salinity and heavy metal stresses. Na and Cd uptake by bacterial cells is presented in Table 6. These results validate the detoxifying role of PGPR. The removal efficiency of Na and Cd by bacterial strain B11 was 67.08% and 45.24%, respectively.

**TABLE 6:** Macronutrient, Na, and Cd accumulation in pepper plants and bacterial cells grown under stress and control conditions with or without PGPR.

Sample name	Ca (μg/kg)	K (μg/kg)	P (μg/kg)	Na (μg/kg)	Cd (μg/kg)
**8DAT-Plant**					
Cont	6.45 ± 0.05g	41.38 ± 0.58h	5.01 ± 0.01h	3.13 ± 0.03e	ND
B11	8.38 ± 0.18d	49.64 ± 0.50g	6.76 ± 0.23c	4.10 ± 0.10c	ND
S	12.88 ± 0.11a	54.11 ± 1.0e	5.31 ± 0.11g	13.74 ± 0.26a	ND
S+B11	8.81 ± 0.31c	63.35 ± 2.05a	6.68 ± 0.22d	8.88 ± 0.38b	ND
Dr	7.94 ± 0.05f	50.38 ± 0.38f	5.40 ± 0.20f	3.10 ± 0.10f	ND
Dr+B11	6.28 ± 0.08h	55.71 ± 0.99d	6.80 ± 0.30b	4.10 ± 0.10c	ND
Cd	11.06 ± 0.13b	55.81 ± 0.50c	5.50 ± 0.20e	3.25 ± 0.05d	1.65 ± 0.35a
Cd+B11	8.11 ± 0.11e	57.63 ± 1.0b	7.73 ± 0.26a	4.10 ± 0.10c	0.52 ± 0.02b

**Sample name**	**Ca (mg/L)**	**K (mg/L)**	**P (mg/L)**	**Na (mg/L)**	**Cd (mg/L)**

**Bacterial cell**					
B11	1.69 ± 0.03c	237.72 ± 1.07b	70.93 ± 0.46a	165.53 ± 0.19b	ND
S+B11	1.94 ± 0.20a	378.81 ± 0.27a	67.53 ± 0.27c	3,462.91 ± 8.43a	ND
Cd+B11	1.90 ± 0.02d	234.55 ± 1.55d	58.02 ± 0.12b	163.81 ± 0.90d	631.10 ± 9.43a
PEG+B11	1.42 ± 0.04b	214.26 ± 0.86c	67.57 ± 0.86d	145.45 ± 1.33c	ND

*Values show the means ± SE (n = 6) and significant differences at p < 0.05 (LSD test). LSD, least significant difference; PEG, polyethylene glycol; PGPR, plant growth-promoting rhizobacteria.*

### Effect of Plant Growth-Promoting Rhizobacteria Treatment on the Expression of Salt-, Drought-, and Heavy Metal-Responsive Genes

Pepper seedlings were used to study the expression of abiotic stress-responsive genes. In total, eight genes (Supplementary Table 2) were analyzed for their change in expression in pepper plant seedlings under abiotic stresses and PGPR inoculation (B11).

### Expression of Genes Related to Binding Immunoglobulin Proteins

**FIGURE 5:**
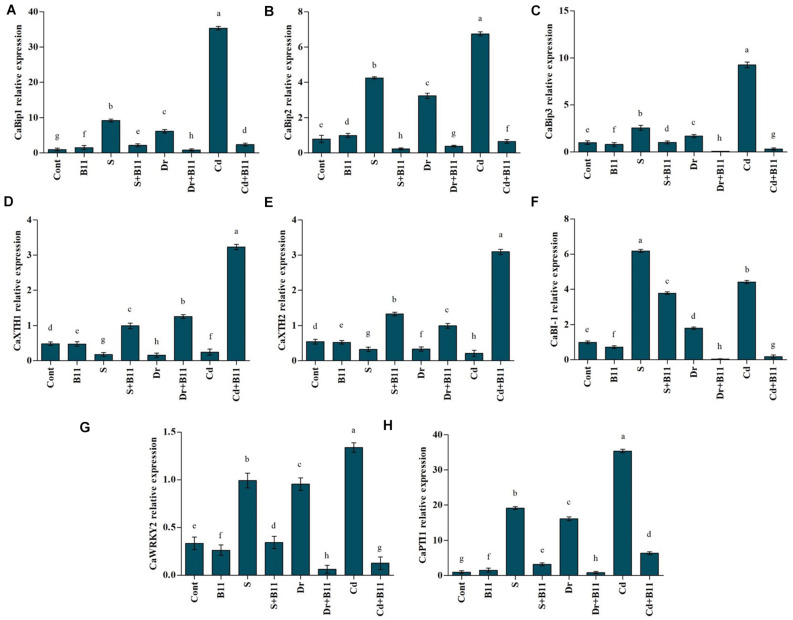
Real-time expression analysis of CaBiPs (*CaBiP1*, **A**; *CaBiP2*, **B**; and *CaBiP3*, **C**), CaXTHs (*CaXTH1*, **D**; and *CaXTH2*, **E**), *CaBI-1* **(F)**, *CaWRKY2* **(G)**, *CapTT1*, and **(H)** in leaves of pepper grown under normal and stress conditions and treated with plant growth-promoting rhizobacteria (PGPR) after 8 days (8DAT). Treatment: control + water, control + PGPR, control + 1% NaCl, PGPR + 1% NaCl, control + 10% polyethylene glycol (PEG), PGPR + 10% PEG, control + 0.1% Cd, and PGPR + 0.1% Cd. Values show the means ± SE (*n* = 3) and significant differences at *p* < 0.05 [least significant difference (LSD) test].

This study characterized three binding immunoglobulin protein (BiP) genes (*CaBiP1*, *CaBiP2*, and *CaBiP3*) in pepper plants. These genes showed differential responses in pepper seedlings under abiotic stress and PGPR inoculation. Salt-, drought-, and heavy metal-stressed plants showed an increase in *CaBiP1* expression levels relative to expression in unstressed control plants; however, among salt-, drought-, and heavy metal-stressed plants, the PGPR inoculation reduced expression by 76.30, 86.19, and 93.37%, respectively, compared with that in uninoculated plants (Figure 5A). Additionally, abiotic stresses enhanced *CaBiP2* gene expression in uninoculated stressed plants in comparison with inoculated control plants. PGPR-inoculated salt-, drought-, and heavy metal-stressed plants showed lower *CaBiP2* gene expression relative to the expression in uninoculated stressed plants (Figure 5B). *CaBiP3* expression levels were increased significantly in heavy metal-stressed plants in comparison with control plants; however, under heavy metal stress conditions, *CaBiP3* expression reduced remarkably (96.53%) in PGPR-inoculated stressed plants (Figure 5C). Furthermore, PGPR-inoculated salt-stressed plants showed a 60.22% decrease in *CaBiP3* expression compared with expression levels in uninoculated salt-stressed plants.

### Expression of Genes Related to Xyloglucan Endotransglucosylase/Hydrolase

The quantitative expressions of *CaXTH1* and *CaXTH2* genes in pepper seedlings under abiotic stresses and PGPR inoculation are shown in Figures 5D,E. Application of abiotic stresses reduced *CaXTH* gene expression in pepper plants, whereas the expression of these genes increased in PGPR-treated plants. For instance, PGPR exposure enhanced *CaXTH2* gene expression by approximately 75.69%, 66.64%, and 93.26% under salinity, drought, and heavy metal stresses compared with the respective expressions in stressed plants alone.

### Expression of Genes Related to BAX Inhibitor 1 (*CaBI-1*)

The effects of salinity, drought, and heavy metal stresses as well as PGPR inoculation on BAX inhibitor 1 (*BI-1*; anti-PCD) was studied in pepper seedlings through the change in the expression of the *BI-1* gene (*CaBI-1*) (Figure 5F). Expression levels of this gene in control and PGPR-inoculated plants displayed minor differences under unstressed conditions; however, higher *CaBI-1* expression was noticed in stressed plants. PGPR-inoculated plants showed a decrease in *CaBI-1* expression compared to uninoculated stressed plants (38.79% under salinity, 97.40% under drought, and 95.79% under heavy metal stress conditions).

### Expression of Transcription Factor *WRKY2*

The expression of the gene related to the transcription factor *WRKY2* (*CaWRKY2*) was also examined. Salinity-, drought-, and heavy metal-stressed plants exhibited a 66.35%, 65.03%, and 75.03% increase, respectively, in *CaWRKY2* expression levels compared with the expression observed in unstressed plants. However, PGPR-inoculated stressed plants showed a decrease in gene expression levels relative to the expression recorded in uninoculated stressed plants. *CaWRKY2* levels in stressed pepper plants were reduced in PGPR-treated plants in comparison to uninoculated stressed plants (64.06% under salinity, 93.74% under drought, and 90.61% under heavy metal stresses; Figure 5G).

### Expression of Genes Related to Ethylene (*CaPTI1*)

The expression level of *CaPTI1* gene was evaluated in pepper seedlings subjected to abiotic stresses and PGPR inoculation. As shown in Figure 5H, salinity-, drought-, and heavy metal-stressed plants demonstrated a 94.82%, 93.84%, and 97.19% rise, respectively, in *CaPTI1* expression levels in comparison with the expression detected in unstressed plants while reduced expression level was observed in PGPR-inoculated stressed plants. PGPR treatment lowered the *CaPTI1* expression level by 83.44% under salinity, 94.75% under drought, and 82.07% under heavy metal stresses compared to the uninoculated stressed plants (Figure 5H).

## Discussion

Plants and bacteria have established synergistic interactions to suppress deleterious stresses (Khan et al., 2019; Pandey and Gupta, 2019). The use of PGPR as a bioinoculant is a promising tool for crop improvement (Pan et al., 2019). For example, PGPR is capable of solubilizing phosphate and releasing different chelating agents; hence, it influences the movement and accessibility of various metabolites in the rhizosphere and mediates the process of phytoremediation and nutrient recycling. Additionally, PGPR can also function in plant growth, management of plant diseases, remediation of hazardous substances, and improvement of soil structure and fertility (Jing et al., 2007; Tangahu et al., 2011; Oves et al., 2017). Plant microbe cooperation provides a specific source of carbon to bacteria that allows them to mitigate the toxicity of polluted regions (Khan and Bano, 2016).

Our results demonstrate that stressed seedlings inoculated with PGPR maintain higher growth parameters in contrast to stressed plants without PGPR treatment. We observed increases in chlorophyll and carotenoid contents in the leaves of PGPR-treated plants under stress conditions. Moreover, we found that PGPR treatment increased K and P contents and reduced Ca, Na, and Cd levels relative to the effects observed in uninoculated stressed plants. Previous studies have indicated that PGPR inoculation boosts the photosynthetic pigments in plants under stress conditions (Izanloo et al., 2008; Bhattacharyya and Jha, 2012). This could be attributed to the higher accessibility and uptake of nutrients from the rhizosphere, which helps maintain plant growth under stress conditions (Çakmakçı et al., 2007; Pii et al., 2015).

Xyloglucan endotransglucosylase/hydrolases (XTHs), as cell growth promoters, play a crucial role in plant development. Moreover, they are involved in plant responses to environmental stimuli including salinity (Cho et al., 2006), water deficit (Choi et al., 2011), heat, cold (Xu et al., 1995), and flood (Saab and Sachs, 1995). In the current study, reduced XTH gene expression was observed in stressed plants, which likely led to decreased cell wall extensibility and retardation in pepper seedlings. In contrast, PGPR-treated stressed plants showed higher XTH gene expression, which led to enhanced plant height and leaf length/width. It has been shown that overexpression of XTH1 and XTH3 enhances salt and drought tolerance in tobacco and pepper plants (Choi et al., 2011; Han et al., 2013). Our results show that PGPR application ameliorates the expression of XTHs (*CaXTH1* and *CaXTH2*) and improves abiotic stress tolerance in pepper seedlings.

Phytohormones regulate the growth and development of plants and help plants acclimate under environmental challenges (Zhang et al., 2006; Yazdanpanah et al., 2011). SA improves photosynthetic and growth parameters in plants and antagonizes oxidative damage in plants in response to abiotic stress (Nazar et al., 2011; Wani et al., 2016). ET is engaged in plant growth, fruit ripening, and leaf senescence along with plant reaction to abiotic and biotic stresses (Gamalero and Glick, 2012). Previous studies have reported the impact of abiotic stress on phytohormone content, including that of SA, ET, and ABA (Yang et al., 2017; Riyazuddin et al., 2020). Yasuda et al. (2008) indicated that antagonistic crosstalk exists between SA and ABA signaling. The present study results indicate that abiotic stresses increase ABA and ET contents but reduce SA levels, which is in accordance with previous reports (Madhaiyan et al., 2006; Khan et al., 2013; Ryu and Cho, 2015; Wu et al., 2017). Our findings show that treatment with PGPR improves stress tolerance in pepper seedlings by lowering the ABA and ET contents and elevating the SA content.

WRKY, one of the largest transcription factor families, is involved in various developmental and physiological activities including abiotic and biotic stress signaling pathways (Huang and Duman, 2002; Seki et al., 2002; Rizhsky et al., 2004; Oh et al., 2006; Qiu and Yu, 2009; Cheng et al., 2016). Analysis of WRKY expression in the current study revealed its high expression under abiotic stress conditions, which was consistent with a previous report (Pandey and Somssich, 2009). Several WRKY transcription factors have been found to function in ABA and SA signaling pathways (Jiang and Yu, 2009; Jiang and Yu, 2015). ABA and SA also show antagonistic interactions toward each other in response to abiotic stresses, which is supported by our results (Yasuda et al., 2008). One previous study indicated that ABA negatively or positively controls the transcripts of some WRKYs (Xie et al., 2005). We observed that *WRKY2* transcript levels were higher in pepper plants exposed to abiotic stresses in the presence of ABA. On the other hand, when ABA levels were reduced in PGPR-treated stressed plants, the expression level of *WRKY2* decreased. These observations demonstrate that ABA positively mediates the expression of *WRKY2* in pepper plants. Taken together, our findings related to *WRKY2* (*CaWRKY2*), ABA, and SA levels in PGPR-treated plants allow us to conclude that PGPR helps stressed plants to cope with various abiotic stresses.

Unfavorable environmental conditions have harmful effects on plant growth and development, and they can trigger protein denaturation or misfolding (Wang et al., 2017). Endoplasmic reticulum stress is activated by misfolded proteins that accumulate in the endoplasmic reticulum under adverse environmental conditions and ultimately result in program cell death (Howell, 2013; Mishiba et al., 2013). Accumulation of misfolded proteins leads to upregulation of BiP genes, which prevent protein augmentation and promote plant tolerance to abiotic and biotic stresses (Mori, 2000). BiPs play key roles in protein quality control by identifying and refolding misfolded proteins (Yang et al., 2016; Wang et al., 2017). In the present study, biochemical/molecular analyses revealed that stressed plants showed increased protein content by the end of the experiment. BiP genes (*CaBiP1*, *CaBiP2*, and *CaBiP3*) were highly expressed due to salinity, drought, and heavy metal stress conditions. This could have been due to an accumulation of unfolded proteins, which may have led to the enhanced induction of BiPs. On the other hand, BiPs were repressed in PGPR-treated plants subjected to stress conditions. Thus, our findings show that PGPR reduces the soluble protein content of stressed plants, which may be due to the stress-relieving effect of PGPR and consequent protein catabolism.

With environmental stresses, plants produce reactive oxygen species (ROS), which at high levels can cause oxidative damage, impair membrane lipid functions, inactivate enzymes, and impede metabolic activities (Huang et al., 2019). In the present study, we observed that H_2_O_2_ and MDA levels were clearly higher in stressed plants, which agrees with a previous report (Batool et al., 2020). PGPR application, however, obviously mitigated the high H_2_O_2_ and MDA levels in stressed plants by the end of the experiment. Therefore, PGPR might suppress the production of ROS and thereby prevent oxidative-based cell membrane damage under environmental stress (Han and Lee, 2005; Singh et al., 2015; Banik et al., 2016; Batool et al., 2020).

Reactive oxygen species are known to be key players in programmed cell death (PCD), a cell suicide process that eliminates damaged cells. BCL2-associated x protein (BAX) has been implicated as an important regulator of PCD and is balanced by the activity of an anti-PCD factor, *BI-1* (Hückelhoven, 2004). In pepper plants, *CaBI-1* expression is induced in response to several types of environmental stresses, such as cold, salinity, drought, submergence, and heavy metal stress, and it provides tolerance to plants toward these stresses (Isbat et al., 2009; Jaiswal et al., 2019). Increased expression of *CaBI-1* was detected in the stressed pepper plants studied here, which is in agreement with the findings of a previous study (Isbat et al., 2009). However, we observed reduced *CaBI-1* expression in stressed plants treated with PGPR. This finding confirms the efficiency and ameliorating effect of PGPR in pepper plants subjected to salinity, drought, and heavy metal stresses.

To cope with oxidative damage and reduce excessive ROS accumulation, plants have developed defensive mechanisms that involve antioxidants with enzymatic or nonenzymatic activities (Agarwal and Pandey, 2004). Several studies have demonstrated that PGPR boosts the activity of enzymatic/nonenzymatic antioxidants. In the present study, the activity of antioxidants increased in stressed plants, but this activity was reduced following PGPR treatment in these abiotically stressed plants. This decrease in antioxidant activity indicates that PGPR improves the ability to scavenge excessive ROS and reduces oxidative damage, which contributes to the protection of photosynthetic processes (Banik et al., 2016).

Sugar sustains macromolecule and membrane structure during drastic water loss in plants; environmental stress can lower leaf sugar content and thereby lead to physiological and biochemical changes (Fenando et al., 2000). It has been demonstrated that an accumulation of soluble sugars mediated by PGPR produces drought resistance in plants because the soluble sugars or sugar by-products function as osmoprotectants under water-deficit conditions (Sánchez et al., 1998). In the current study, a clear increase in leaf sugar content was apparent in pepper seedlings treated with PGPR under normal conditions and stress conditions. Soluble sugars also function as metabolic resources and structural constituents of cells, and they modulate many processes associated with plant development under water stress conditions (Wang and Ruan, 2013). Sugar accumulation in the leaf also triggers the expression of genes connected to photosynthetic activities (Van Oosten and Besford, 1995). In our work, PGPR treatment induced higher sugar accumulation, which potentially acted as an osmoprotectant in photosynthetic organs and helped maintain the photosynthetic efficiency of the plants, resulting in their increased growth under stress conditions.

Amino acids directly or indirectly regulate plant responses to environmental stresses (Ashraf and Harris, 2004). In our experiment, abiotic stresses increased the amino acid content in pepper seedlings. This accumulation suggests that the amino acids play a role in osmotic adjustment as well as in metabolism (Wu et al., 2014). Previous reviews have reported that amino acid content increases in plants under stress (Rare, 1990; Mansour, 2000). The application of PGPR rescued amino acid content in many cases in stressed seedlings during the recovery period. A rapid increase in proline content was observed in stressed seedlings, which is in agreement with the findings of previous studies on other plant species (Khattab, 2007; Bassuony et al., 2008). Accumulation of proline, which functions as osmoprotectant and ROS scavenger, could be one method by which plants endure abiotic stress (Ghars et al., 2008; Dawood and El-Awadi, 2015; Kim et al., 2016; Liu et al., 2018). Indeed, increased proline content has been associated with enhanced plant performance under environmental stresses (Nanjo et al., 1999; Khan et al., 2013). Furthermore, proline may play a role as a reservoir of organic nitrogen that can be consumed during the recovery period to help plants withstand environmental challenges (Rare, 1990; Sairam and Tyagi, 2004). In the present study, application of PGPR led to enhanced proline content in stressed seedlings; this PGPR-induced proline increase could represent an adaptive mechanism that facilitates osmotic adjustment and improves plant survival under stress conditions.

## Conclusion

Application of rhizospheric *B. amyloliquefaciens* B11 not only augmented pepper growth under salinity, drought, and metal stresses but also undoubtedly urged pepper endurance to these abiotic stresses. The ability of *B*. *amyloliquefaciens* B11 to solubilize nutrients and secrete IAA, ACC, and siderophore under abiotic induced stress regulated host growth *via* alleviating salt and metal accumulation in pepper plant. Furthermore, *B*. *amyloliquefaciens* B11 inoculation modified the regulation of host antioxidant system and hormones to minimize the destructive effects of abiotic stresses. Inoculation of *B*. *amyloliquefaciens* B11 induced the expression of stress-related genes, namely, CaXTHs, *CaWRKY2*, *CaBI-1*, CaPTI1, and CaBiPs. Taken together, our results provided clear evidence to demonstrate the stress-relieving effect of *B*. *amyloliquefaciens* B11 and propose it as an appropriate candidate for remediation of heavy metal-polluted soils.

## Data Availability Statement

The datasets presented in this study can be found in online repositories. The names of the repository/repositories and accession number(s) can be found in the article/Supplementary Material.

## Author Contributions

EK planned the research, conducted the experiments, analyzed the data, and wrote the manuscript. AA and L-RK helped to conduct the experiments. SM analyzed the sequences. SM and AA-S edited the manuscript. S-MK and I-JL provided the resources. All authors approved the manuscript.

## Conflict of Interest

The authors declare that the research was conducted in the absence of any commercial or financial relationships that could be construed as a potential conflict of interest.

## Supplementary Material

Supplementary Figure 1 **(A)** IAA production, **(B)** ACC deaminase activity, **(C)** siderophore production, **(D)** nitrogen fixation, **(E)** citrate utilization, **(F)** potassium solubilization, **(G)** zinc solubilization, **(H)** phosphate solubilization, and **(I)** silicon solubilization.

Supplementary Figure 2 **(A)** Salt and **(B)** drought tolerance ability of the selected bacterial strain (B11) associated with *Sasamorpha* in this study.

Supplementary Figure 3 Heavy metal tolerance ability of the selected bacterial strain (B11) associated with *Sasamorpha* in this study **(A–C)**.

Supplementary Figure 4 Effects of various salt concentrations on the growth of pepper seedlings following 8 days of treatment (8DAT; **A,B**). Treatments: control + water, 0.5% NaCl, 1% NaCl, 1.5% NaCl, and 2.5% NaCl.

Supplementary Figure 5 Effects of PGPR inoculation on pepper plant growth under normal and stress conditions after 8 days of treatment (8DAT). Treatments: control + water, control + PGPR, control + 1%NaCl, PGPR + 1%NaCl, control + 10%PEG, PGPR + 10%PEG, control + 0.1%Cd, and PGPR + 0.1%Cd. Values show the means ± SE (*n* = 3) and significant differences at *p* < 0.05 (LSD test).
