# Supplementary figures and images for: Rhizospheric Bacillus amyloliquefaciens Protects Capsicum annuum cv. Geumsugangsan From Multiple Abiotic Stresses via Multifarious Plant Growth-Promoting Attributes
Source: Front Plant Sci. 2021 May 25;12:669693. doi: 10.3389/fpls.2021.669693 (PMC8185346; doi:10.3389/fpls.2021.669693)

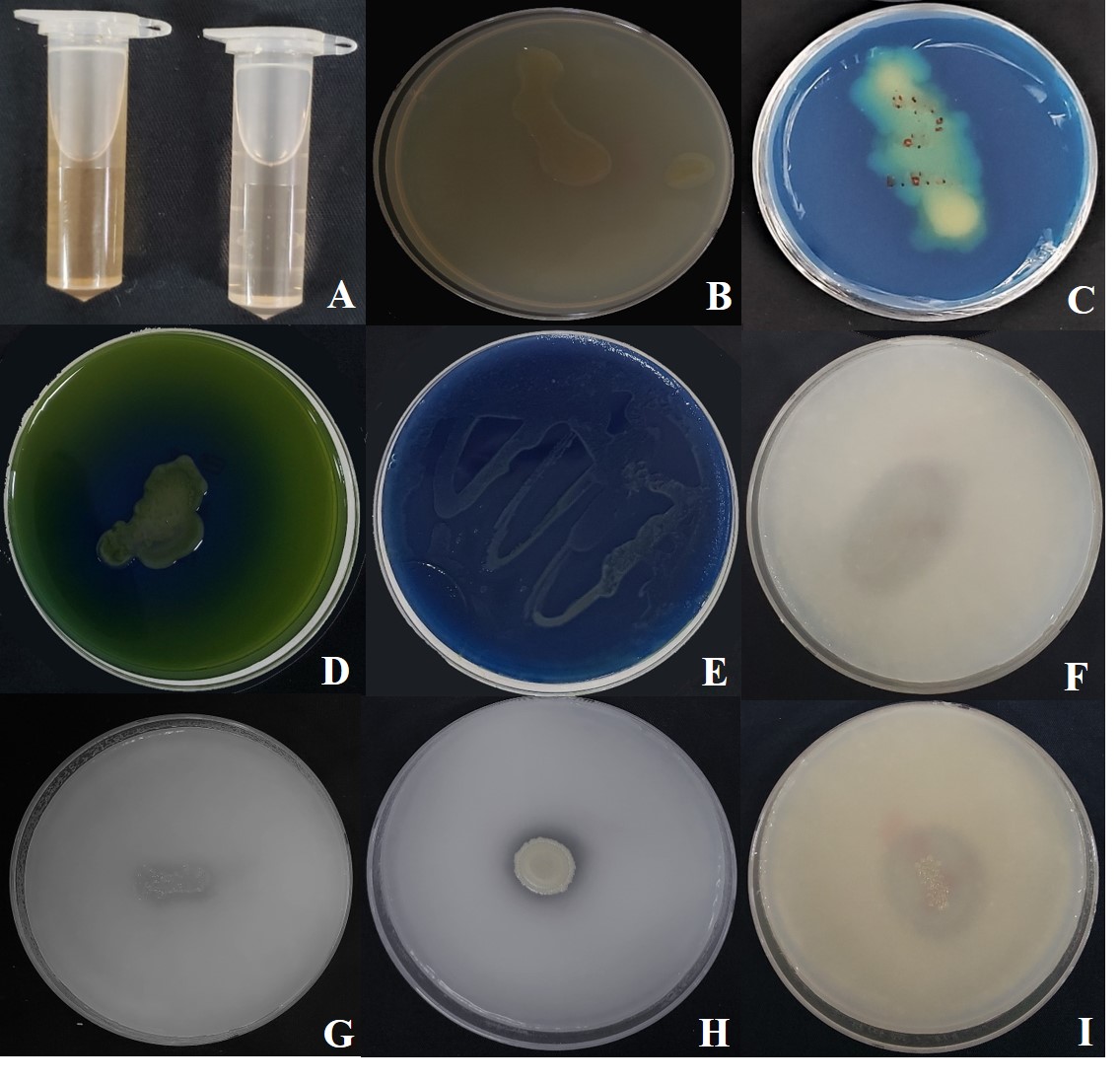

Supplement: Supplementary Figure 1 — (A) IAA production, (B) ACC deaminase activity, (C) siderophore production, (D) nitrogen fixation, (E) citrate utilization, (F) potassium solubilization, (G) zinc solubilization, (H) phosphate solubilization, and (I) silicon solubilization. [file Data_Sheet_1.zip › Supplementary Figure S1.JPEG]

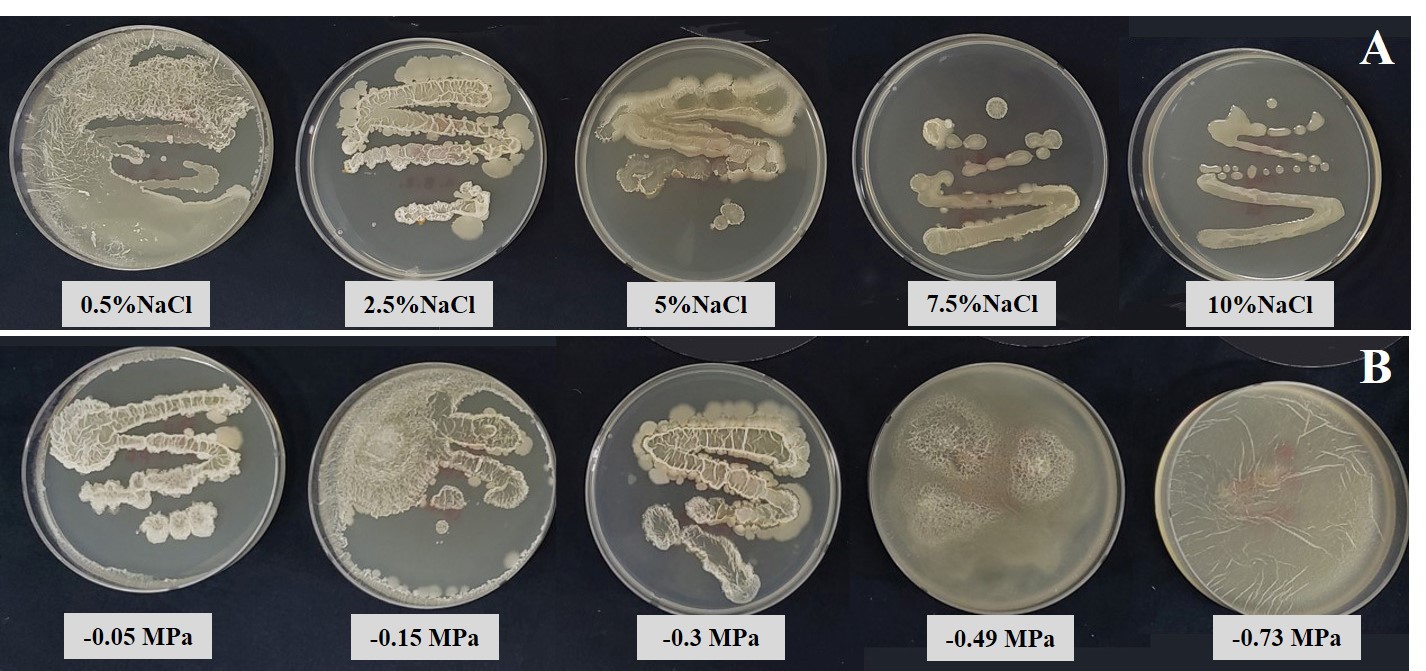

Supplement: Supplementary Figure 1 — (A) IAA production, (B) ACC deaminase activity, (C) siderophore production, (D) nitrogen fixation, (E) citrate utilization, (F) potassium solubilization, (G) zinc solubilization, (H) phosphate solubilization, and (I) silicon solubilization. [file Data_Sheet_1.zip › Supplementary Figure S2.JPEG]

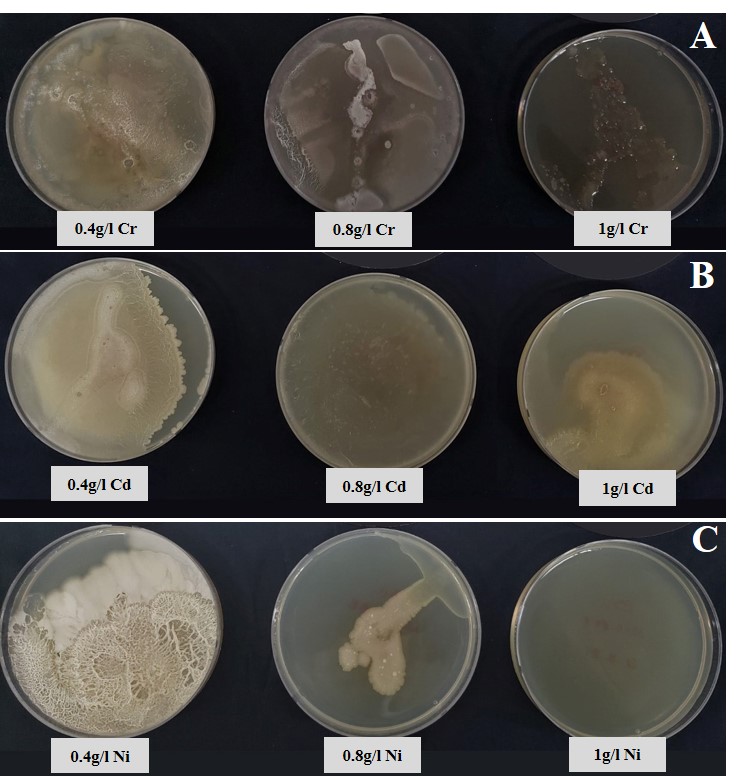

Supplement: Supplementary Figure 1 — (A) IAA production, (B) ACC deaminase activity, (C) siderophore production, (D) nitrogen fixation, (E) citrate utilization, (F) potassium solubilization, (G) zinc solubilization, (H) phosphate solubilization, and (I) silicon solubilization. [file Data_Sheet_1.zip › Supplementary Figure S3.JPEG]

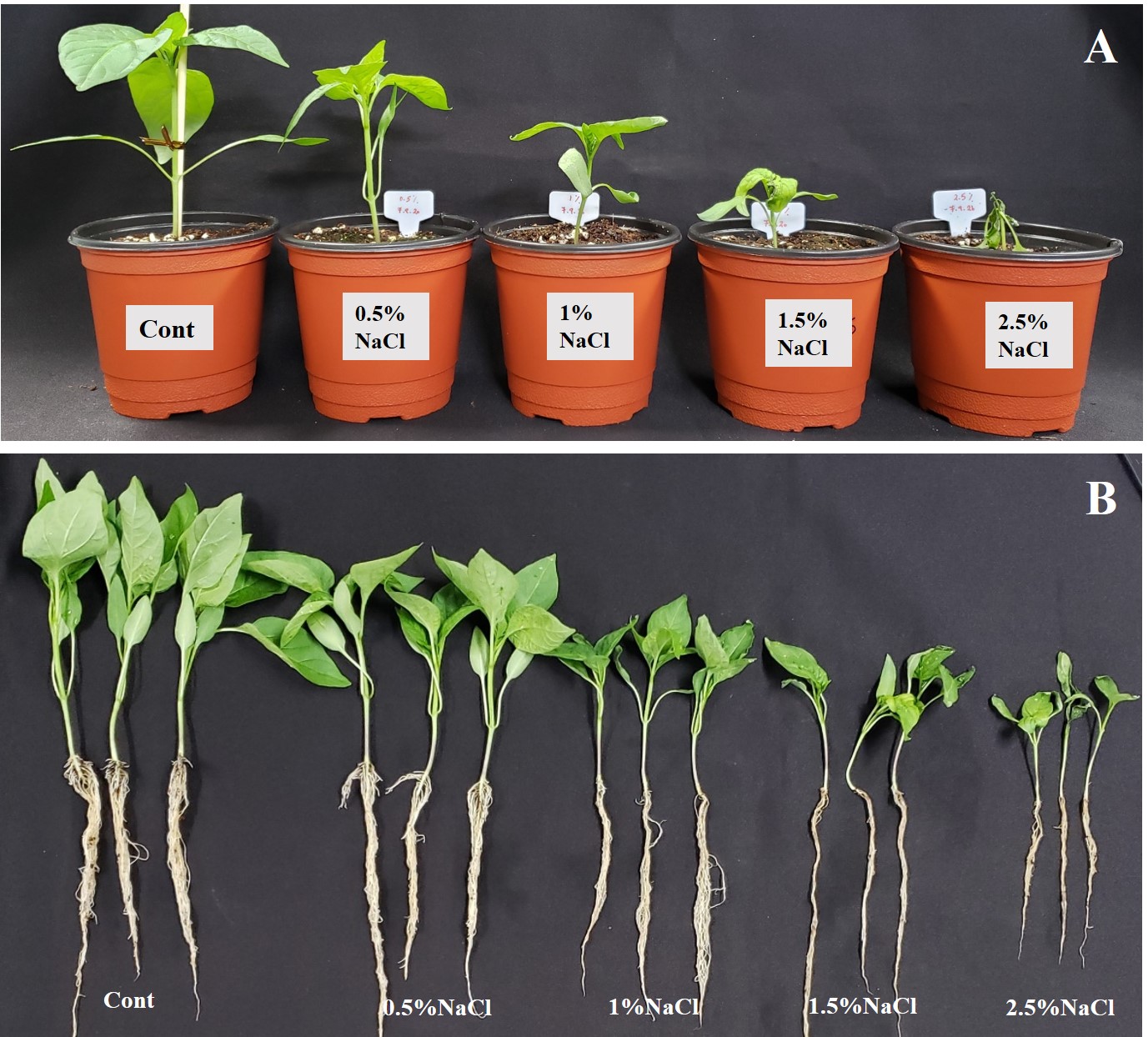

Supplement: Supplementary Figure 1 — (A) IAA production, (B) ACC deaminase activity, (C) siderophore production, (D) nitrogen fixation, (E) citrate utilization, (F) potassium solubilization, (G) zinc solubilization, (H) phosphate solubilization, and (I) silicon solubilization. [file Data_Sheet_1.zip › Supplementary Figure S4.JPEG]

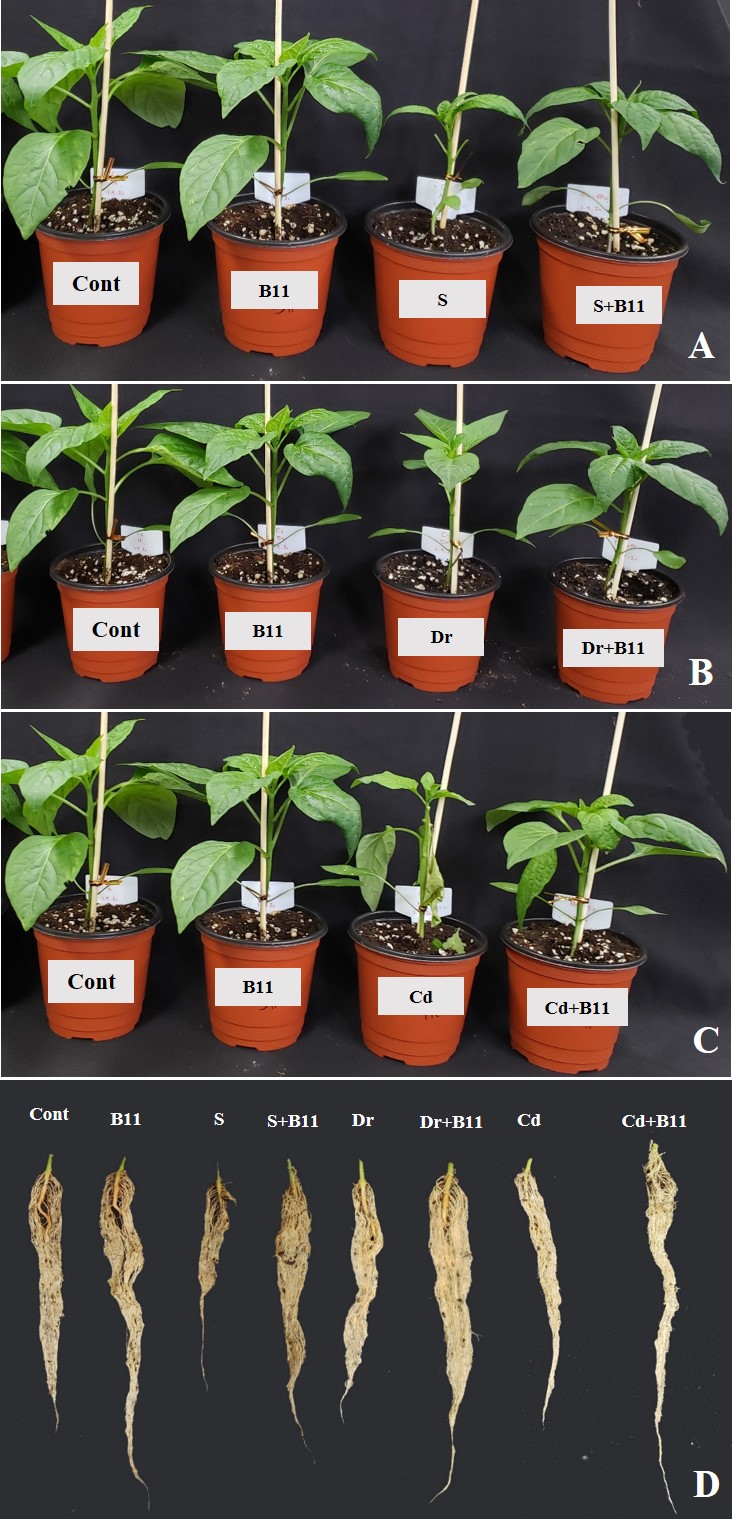

Supplement: Supplementary Figure 1 — (A) IAA production, (B) ACC deaminase activity, (C) siderophore production, (D) nitrogen fixation, (E) citrate utilization, (F) potassium solubilization, (G) zinc solubilization, (H) phosphate solubilization, and (I) silicon solubilization. [file Data_Sheet_1.zip › Supplementary Figure S5.JPEG]
